# Genetic, Psychological, and Behavioural Factors Associated with Subtypes of Pain-Related Temporomandibular Disorders

**DOI:** 10.3390/biomedicines13081961

**Published:** 2025-08-12

**Authors:** Marko Zlendić, Ema Vrbanović Đuričić, Koraljka Gall Trošelj, Marko Tomljanović, Kristina Vuković Đerfi, Ivan Alajbeg, Iva Z. Alajbeg

**Affiliations:** 1 Department of Removable Prosthodontics, University of Zagreb School of Dental Medicine, Zagreb 10000, Croatia; mzlendic@sfzg.hr (M.Z.); evrbanovic@sfzg.hr (E.V.Đ.); 2 Laboratory for Epigenomics, Ruđer Bošković Institute, Zagreb 10000, Croatia; troselj@irb.hr (K.G.T.); marko.tomljanovic@irb.hr (M.T.); 3 Laboratory for Personalised Medicine, Ruđer Bošković Institute, Zagreb 10000, Croatia; kvukovic@irb.hr; 4 Department of Oral Medicine, University of Zagreb School of Dental Medicine, Zagreb 10000, Croatia; alajbeg@sfzg.hr; 5 Department of Dentistry, Clinical Hospital Centre Zagreb; Zagreb 10000, Croatia

**Keywords:** biomarkers, single nucleotide polymorphism, temporomandibular joint disorders, arthralgia, myalgia, chronic pain

## Abstract

**Background:** This genetic association study investigated single nucleotide polymorphism (SNP) in interleukin-8 (*CXCL8*) and opiorphin (*OPRPN*) genes, as well as psychological characteristics and oral behaviours, between patients with pain-related temporomandibular disorders (TMDp) and healthy controls. The aim was to examine associations and predictive value of these factors for TMDp subtypes: arthralgia and myalgia. **Methods:** A total of 85 patients with TMDp (arthralgia and/or myalgia) and 85 pain-free controls were included. Diagnoses were established following the Diagnostic Criteria for Temporomandibular Disorders (DC/TMD). All participants completed standardised self-report questionnaires assessing anxiety, depression, and oral behaviours. Buccal swabs were collected for DNA extraction, and SNP genotyping was performed using real-time PCR. Statistical analyses were conducted using dominant and recessive genetic models. Logistic regression models were applied to assess risk factors for each TMDp subtype. **Results:** Participants homozygous for the minor allele (CC genotype) of rs1387964 in *OPRPN* were significantly more prevalent in both arthralgia and myalgia groups compared to controls. Age and female sex predicted TMDp-arthralgia. Predictors of TMDp-myalgia included the CC genotype of rs1387964, age, female sex, anxiety, and depression. **Conclusions:** Genetic background and psychological characteristics were significant predictors of TMDp myalgia, highlighting a multifactorial profile for this TMDp subtype.

## 1. Introduction

Pain-related temporomandibular disorders (TMDp) are chronic orofacial pain conditions affecting masticatory muscles and temporomandibular joints (TMJ). The Diagnostic Criteria for Temporomandibular Disorders (DC/TMD) classify TMDp into two primary categories: pain-related and joint-related disorders [1]. Unlike acute pain, which serves a protective biological function, chronic pain lacks such a purpose and is instead associated with substantial psychological, social, and physical burdens that impair daily functioning [2]. However, the aetiological mechanisms underlying chronic painful TMD remain poorly understood. It is hypothesised that two distinct phenotypes, heightened pain sensitivity and diminished ability to cope with psychological stress, underlie individuals’ vulnerability to the onset and persistence of TMDp [3]. Adding complexity to this model is the contribution of individual genetic background. Genetic variations, such as single nucleotide polymorphisms (SNPs), may influence these phenotypes and thus modulate pain perception [4]. 

One of the proinflammatory cytokines implicated in chronic pain is interleukin-8 (IL-8), encoded by the gene C-X-C motif chemokine ligand 8 (*CXCL8*). Distinct inflammatory profiles between patients with acute and chronic pain suggest that proinflammatory mediators may play differential roles depending on pain duration [5]. Persistent elevation of cytokines such as IL-8 has been proposed to contribute to altered pain perception and central sensitisation [6]. Supporting this, altered endogenous pain modulation has been observed in patients with rheumatoid arthritis, a chronic immune-mediated disorder [7]. Reduced pain inhibition or enhanced pain excitation leads to central sensitisation, a key feature of TMDp [8]. In our previous research, the minor G allele of SNP rs2227307 in *CXCL8* was associated with an increased risk of TMDp and predicted higher pain intensity among carriers [9]. 

Another potential contributor to the sensitisation process is opiorphin. Salivary opiorphin levels are elevated in both acute and chronic pain conditions and could serve as a valuable biomarker in several oral disorders [10,11]. Elevated levels of opiorphin have been observed in patients with burning mouth syndrome, a chronic orofacial condition of unclear aetiology [12]. Similarly, increased salivary concentrations have been reported in patients with acute dental pulp inflammation [13]. As opiorphin exhibits antinociceptive activity, an imbalance in its function could contribute to pain chronicity. In our previous study, individuals homozygous for the minor allele of rs1387964 in the opiorphin gene (*OPRPN*) were more frequently represented in the TMDp group, and this genotype was predictive of TMDp presence [14]. 

Psychological characteristics, particularly elevated levels of anxiety and depression, are well-documented risk factors for chronic pain disorders [15,16]. TMD patients exhibited higher levels of psychological distress, increased frequency of both waking-state and sleep-related oral behaviours, and greater somatosensory amplification compared to healthy controls [17,18]. In addition to being more frequent in TMDp patients, oral behaviours were also more prevalent among TMD patients with greater pain intensity than those with milder symptoms [18,19]. 

Despite this growing body of evidence, the complex relationship between TMDp subtypes (arthralgia and myalgia) and interacting factors, such as genetic background, psychological characteristics, and oral behavioural habits, remains insufficiently understood. Therefore, the aim of this study was to examine potential associations between SNP genotypes in *CXCL8* and *OPRPN*, psychological characteristics (anxiety and depression), and the frequency of oral behaviours with specific TMDp subtypes. Additionally, we examined the predictive value of these factors in explaining their potential differential impact on TMDp subtypes.

## 2. Materials and Methods

### 2.1. Study Design

This case–control genetic association study was conducted in Croatia, at the University of Zagreb School of Dental Medicine, the Department of Dental Medicine at the University Hospital Centre Zagreb, and the Ruđer Bošković Institute. This study followed the Strengthening the Reporting of Genetic Association Studies (STREGA) guidelines to improve the overall quality and transparency of the reported findings [20]. The study complied with the ethical standards of the Declaration of Helsinki and was approved by the Ethics Committee of the University of Zagreb School of Dental Medicine (approval number: 05-PA-30-VIII-6/2019). The clinical trial was registered on 5 January 2021 at ClinicalTrials.gov (identifier: NCT046).

### 2.2. Enrolment of Participants and Eligibility Criteria

Enrolment of participants began in January 2020 and was completed in September 2022. During this period, a total of 341 patients were referred to the Department of Dental Medicine at the University Hospital Centre Zagreb and were examined by calibrated and experienced orofacial pain experts (IZA, EVĐ, MZ). Out of the total, 85 (76 females and 9 males) met the inclusion criteria and agreed to participate. Additionally, 85 (62 females and 23 males) healthy control participants were enrolled. Prior to enrolment, all participants signed an informed consent form that contained all relevant information about the study.

Inclusion criteria for the case group (TMDp patients) were age  ≥  18 years; reported pain in the TMJ and/or masticatory muscles persisting or recurring for more than three months; and pain intensity greater than 30 mm on the Numerical Pain Rating Scale (NPRS) at the time of the initial examination.

Exclusion criteria for the case group (TMDp patients) were age  <  18 years; orofacial pathology unrelated to the TMD diagnosis, such as periodontitis; acute pain (i.e., pain present for less than three months); pain caused by fibromyalgia, systemic diseases, or diagnosed psychiatric disorders; and a history of, or current, pain medication abuse. Participants who exhibited painless joint sounds and/or jaw locking were also excluded from the study.

The control group comprised adult volunteers (≥ 18 years) in good general health, with no current or previous chronic pain disorders affecting any part of the body, and no current use of analgesic medication. This group included employees of the Department of Dental Medicine at the Clinical Hospital Centre Zagreb, as well as employees and students of the University of Zagreb School of Dental Medicine.

All participants were Caucasian, with a Middle or Southern European background. The sample size calculation for a case–control study with an equal participant ratio (1:1) was based on an estimated overall TMD prevalence of approximately 5%, as reported in the literature. The calculation assumed a significance level of 5% (α = 0.05) and a power of 80% under a dominant inheritance model. Accordingly, a total of 150 participants (75 patients with TMD and 75 healthy controls) would be required to achieve sufficient statistical power [21,22].

### 2.3. Assessment of TMDp Patients

To be included in the case group, participants were required to be diagnosed with TMDp, presenting with muscle pain (local myalgia, myofascial pain, or myofascial pain with referral) and/or joint pain (arthralgia), in accordance with the DC/TMD protocol. Data were collected through patient history, clinical examination, and validated questionnaires, such as the Graded Chronic Pain Scale (GCPS), to confirm pain chronicity. 

The patient history included the reason for seeking professional help, pain characteristics (duration, intensity, and type), functional limitations of the mandible, current pharmacotherapy, and awareness of oral behavioural habits. The clinical examination included confirmation of the pain location; palpation of the masticatory muscles, TMJs, and the submandibular and retromandibular regions; measurement of mandibular movements (pain-free mouth opening [MO], unassisted MO, assisted MO, lateral movements, and protrusion); and assessment of the presence and characteristics of TMJ sounds [1]. Dental status was assessed, and panoramic radiographs were reviewed. 

Participants were subsequently provided with standardised instructions and asked to independently complete the following questionnaires: the Generalised Anxiety Disorder-7 (GAD-7), the Patient Health Questionnaire-9 (PHQ-9), and the Oral Behaviours Checklist (OBC) [1].

### 2.4. Psychological Characteristics of Participants

Assessment of participants’ psychological characteristics, specifically anxiety and depression, was carried out using the GAD-7 and PHQ-9 questionnaires. Both questionnaires are validated for use in the general population and are part of the self-report instrument set within Axis II of the DC/TMD protocol [1,23,24]. 

The GAD-7 is a seven-item questionnaire used to assess the severity of anxiety symptoms, while the PHQ-9 is a nine-item instrument designed to screen for depressive symptoms. Both are based on a four-point Likert scale ranging from “0” (not at all) to “3” (almost every day). The possible total scores range from 0 to 21 for the GAD-7 and from 0 to 27 for the PHQ-9.

### 2.5. Frequency of Oral Behaviours 

The frequency of oral behaviours was assessed using the Oral Behaviours Checklist (OBC) questionnaire. This instrument is also part of Axis II of the DC/TMD protocol and has been validated for use in the general population [25,26]. The OBC is a 21-item questionnaire that uses a five-point Likert scale ranging from “none of the time” to “all of the time”. Each item is scored from 0 to 4, yielding a total score range from 0 to 84.

### 2.6. Grouping of Participants

Grouping of TMDp patients was based on the DC/TMD pain-related diagnoses of arthralgia and myalgia. All patients diagnosed with arthralgia, either as a single diagnosis or in combination with myalgia, were assigned to the TMDp arthralgia group (*n* = 74). All patients diagnosed with myalgia, either alone or in combination with arthralgia, were assigned to the TMDp myalgia group (*n* = 55).

All participants were grouped based on the frequency of oral behavioural habits, as measured by the OBC total score. According to the Diagnostic Criteria for Temporomandibular Disorders (DC/TMD) Scoring Manual for Self-Report Instruments by Ohrbach and Knibbe, participants were classified into two groups: the high-frequency parafunction group (OBC sum score 25–84) and the low-frequency parafunction group (OBC sum score 1–24).

All participants were also grouped based on the severity of anxiety symptoms, assessed using the GAD-7 questionnaire. Following the same DC/TMD scoring manual, participants were divided into two groups: the moderate to severe anxiety group (GAD-7 score 10–21) and the mild anxiety group (GAD-7 score 1–9).

Similarly, participants were grouped based on the severity of depressive symptoms, assessed using the PHQ-9. According to the DC/TMD scoring guidelines, participants were divided into two groups: the moderate to severe depression group (PHQ-9 score 10–27) and the mild depression group (PHQ-9 score 1–9).

### 2.7. Selection of Genes and SNPs

The CXC motif chemokine ligand 8 gene (*CXCL8*; Gene ID: 3576) encodes the protein interleukin-8. The gene is located on chromosome 4q13.3 and comprises four exons and three introns. In this research, we examined two SNPs, namely, rs2227306—C_11748169_10 and rs2227307—C_11748168_10, in *CXCL8*.

The polymorphism rs2227306 (NC_000004.12:73741337:C:T) is located in a non-coding region of *CXCL8* and can present as cytosine (C) or thymine (T).

Similarly, polymorphism rs2227307 (NC_000004.12:73740951:T:G) is located in a non-coding region of *CXCL8* and can present as thymine (T) or guanine (G). 

The opiorphin prepropeptide gene (*OPRPN*; Gene ID: 58503) encodes a member of the proline-rich protein family known as opiorphin. The gene is located on 4q13.3 and comprises three exons and two introns. In this study, we examined SNP rs1387964 in *OPRPN*.

The rs1387964—C3201429_10) polymorphism (NC_000004.12:70396196:C:T) is located in the promoter region of *OPRPN* and can present as thymine (T) or cytosine (C).

The selection of the genes and SNPs investigated in this study was based on their involvement in pain processing pathways. Additionally, this research builds upon our previous work and contributes new insights into the role of these genes in chronic pain disorders.

### 2.8. Genetic Analysis

Buccal swabs were collected from all participants using a soft nylon bristle brush (Mirandola, Rimos, Italy). Participants were instructed to refrain from eating or drinking for at least two hours prior to sample collection and to rinse their mouths with plain water immediately before the procedure. The swabs were stored in microtubes filled with phosphate-buffered saline (PBS) (Eppendorf, Hamburg, Germany) and preserved at −20 °C for subsequent DNA extraction.

Genomic DNA was extracted using the QIAamp® DNA Mini Kit (QIAGEN, Venlo, The Netherlands). The quality of extracted DNA was assessed using 1% agarose gel electrophoresis with ethidium bromide staining and was satisfactory for all samples. DNA concentration was determined by measuring absorbance at 260 and 280 nm using a NanoDrop™ spectrophotometer (Thermo Fisher Scientific, Massachusetts, USA). The yield of extracted DNA ranged from 10 to 30 μg/μL, which was sufficient for SNP genotyping.

For each sample, a 20 μL reaction mixture was prepared in 0.2 mL optical tubes (Applied Biosystems, Waltham, MA, USA), consisting of 1 μL DNA, 1 μL TaqMan™ Genotyping Assay (Applied Biosystems), 10 μL TaqPath™ ProAmp™ Master Mix (Applied Biosystems), and 8 μL Milli-Q water. Genotyping was performed using the 7300 Real-Time PCR System (Applied Biosystems) under the following conditions: initial denaturation at 50 °C for 2 min and 95 °C for 10 min, followed by 40 cycles of 15 s at 95 °C and 1 min at 60 °C.

Quality control measures included the use of negative (no template) and positive controls, blind technical duplicates to detect discrepancies, removal of low-quality DNA samples, and evaluation of genotype distributions for deviation from Hardy–Weinberg equilibrium.

### 2.9. Statistical Analysis

Statistical analyses were performed using IBM SPSS Statistics, version 26.0 (IBM Corp, New York, USA). The Shapiro–Wilk test was used to assess the normality of distribution. Group differences in demographic and clinical characteristics were assessed using the independent samples *t*-test and chi-square test. The association between genotype frequency and TMDp subtype (TMDp/CTR) was tested using the chi-square test and Fisher’s exact test after fitting for the Hardy–Weinberg equilibrium. For this purpose, genetic models (dominant and recessive) were constructed.

The assessment for *CXCL8* was conducted using a dominant genetic model, considering the effect of carrying one or both minor alleles. The assessment for *OPRPN* was conducted using a recessive genetic model, considering the effect of carrying both minor alleles. In both models, the major allele served as the reference allele, while the minor allele was identified as the risk allele. Logistic regression analysis was performed to identify factors associated with TMDp subtypes (arthralgia and myalgia).

Values of *p* < 0.05 were considered statistically significant, and the risks associated with pain-related TMD subtypes were calculated as the odds ratio (OR) with a 95% confidence interval (CI).

## 3. Results 

### 3.1. Participant Characteristics

The TMDp group consisted of 85 participants, predominantly women (76 women, 89.4%, and 9 men, 10.6%), with a mean age of 29.9 years (±11.1). Among them, 44 individuals (51.8%) had more than one diagnosis, primarily women (93.2%). Arthralgia was the sole diagnosis in 30 participants (35.3%), again mostly women (93.3%), while 11 individuals (12.9%) had only myalgia, with a lower proportion of women (63.6%). In total, myalgia—whether localised, myofascial, or with referral—was found in 64.7% of the group (55 individuals), and arthralgia was present in 87.1% (74 individuals). 

The control group also included 85 individuals, with 62 women (72.9%) and 23 men (27.1%), and a slightly younger mean age of 26.3 years (±7.7). All members of the control group had no signs of muscle or joint symptoms or other temporomandibular disorders.

A significantly higher proportion of women was observed in the TMDp group compared to the control group; however, the age distribution did not differ significantly. The average duration of pain in the TMDp group was 20.5 months, and more than 58% of participants experienced pain in multiple regions. 

Table 1 presents a comparison of oral behaviours and psychological characteristics between individuals with pain-related temporomandibular disorders (TMDp) and healthy controls, stratified by diagnostic subtype (arthralgia and myalgia). 

According to the Oral Behaviours Checklist, a high frequency of oral behavioural habits was reported in 56.4% of TMDp patients with myalgia, 58.1% of those with arthralgia, and 58.8% of participants in the control group. These differences were not statistically significant.

Moderate to severe anxiety levels (as measured by GAD-7) were observed in 18.2% of patients with myalgia, 17.8% of those with arthralgia, and 5.9% of control participants. The differences between both TMDp subgroups and controls were statistically significant (*p* = 0.022 for myalgia; *p* = 0.019 for arthralgia).

Moderately severe and severe depression (as measured by PHQ-9) was present in 21.8% of participants with myalgia, 19.2% with arthralgia, and 9.4% of controls. A statistically significant difference was found only between the myalgia subgroup and the control group (*p* = 0.040).

### 3.2. Distribution of SNP Genotypes

Table 2 shows the distribution of selected single nucleotide polymorphisms (SNPs) in the *OPRPN* and *CXCL8* genes among individuals with pain-related temporomandibular disorders stratified by TMD subtype (arthralgia and myalgia) and healthy controls.

For the rs1387964 polymorphism in the *OPRPN* gene (recessive model), the frequency of individuals with the homozygous CC genotype was significantly higher in both arthralgia (12.2%) and myalgia (14.5%) groups compared to controls (3.5% in both comparisons). These differences were statistically significant for both subtypes (*p* = 0.040 and *p* = 0.018, respectively), suggesting a potential association between the CC genotype and susceptibility to pain-related TMD, particularly myalgia.

In contrast, for the rs2227306 polymorphism in the *CXCL8* gene (dominant model), no significant differences were observed in genotype distribution between either TMDp subgroup or controls (arthralgia: *p* = 0.084; myalgia: *p* = 0.122). Similarly, the rs2227307 variant in the *CXCL8* gene showed no statistically significant difference; however, the comparison between the arthralgia group and controls approached significance (*p* = 0.052).

### 3.3. Risk Factors Associated with TMDp Diagnosis

A logistic regression model was used to assess the relationship between specific genetic polymorphisms and pain-related TMDs, while accounting for age and sex as possible confounding factors or effect modifiers. Table 3 presents two logistic regression models evaluating potential predictors of arthralgia. Both models include the *CXCL8* rs2227307 polymorphism (dominant model), age, sex, and oral behaviours (OBC-total score). Model I includes anxiety (GAD-7), while Model II includes depression (PHQ-9) as a psychological variable.

In both models, female sex was a strong and significant predictor of arthralgia (Model I: OR = 5.506, *p* = 0.002; Model II: OR = 6.308, *p* = 0.001), indicating substantially higher odds of arthralgia in women. Additionally, age showed a significant positive association in both models (Model I: OR = 1.052, *p* = 0.009; Model II: OR = 1.050, *p* = 0.014), suggesting that the likelihood of arthralgia slightly increases with age.

The *CXCL8* rs2227307 polymorphism was not statistically significant but showed a consistent trend toward increased risk in carriers of the TG or GG genotype (Model I: OR = 1.815, *p* = 0.104; Model II: OR = 1.910, *p* = 0.080). 

Among psychological variables, higher anxiety (GAD-7) was marginally associated with arthralgia in Model I (OR = 3.194, *p* = 0.051), while higher depression (PHQ-9) in Model II also showed a borderline association (OR = 2.728, *p* = 0.069). Oral parafunctional behaviours (OBC) were not significant predictors in either model.

Table 4 displays logistic regression models assessing predictors of myalgia. Both models include the *OPRPN* rs1387964 polymorphism (recessive model), age, sex, and oral behaviours (OBC-total). Model I includes anxiety (GAD-7), and Model II includes depression (PHQ-9).

The rs1387964 polymorphism in the *OPRPN* gene was a strong and significant predictor of myalgia in both models. Individuals with the homozygous CC genotype had markedly higher odds of developing myalgia compared to carriers of the T allele (Model I: OR = 6.638, *p* = 0.011; Model II: OR = 6.738, *p* = 0.012), indicating a potential genetic predisposition linked to this variant.

Age also emerged as a significant predictor in both models (Model I: OR = 1.044, *p* = 0.038; Model II: OR = 1.044, *p* = 0.040), indicating a slight increase in myalgia likelihood with advancing age.

Female sex was significantly associated with myalgia in both models (Model I: OR = 3.158, *p* = 0.033; Model II: OR = 3.748, *p* = 0.016), confirming sex-related vulnerability.

Both anxiety (GAD-7) in Model I (OR = 3.510, *p* = 0.040) and depression (PHQ-9) in Model II (OR = 3.135, *p* = 0.036) were significantly associated with increased odds of myalgia, suggesting a robust contribution of psychological distress.

Oral parafunctional behaviours (OBC) were not significant in either model.

## 4. Discussion

The current study adds to existing knowledge of factors associated with specific pain-related TMD subtypes, myalgia and arthralgia. The influence of genetic background was particularly evident in myalgia; however, both subtypes exhibited some degree of genetically mediated susceptibility. This association was observed in the *OPRPN* gene, where the minor allele homozygous (CC genotype) was more prevalent in both the arthralgia (*p* = 0.040) and myalgia (*p* = 0.025) groups compared to healthy controls. TMDp myalgia showed significant associations with the *OPRPN* rs1387964 variant, female sex, age, and psychological characteristics, highlighting the multifactorial nature of this subtype. In contrast, risk factors for arthralgia were limited to female sex and age, while *CXCL8* polymorphisms and psychological characteristics may play a contributing, albeit nonsignificant, role. Although the frequency of oral behaviours differed significantly between TMDp subtypes and controls, these behaviours did not emerge as significant predictors for either subtype.

The *OPRPN* gene encodes salivary opiorphin, a peptide involved in endogenous antinociceptive mechanisms. In our previous studies, we found that the CC genotype of rs1387964 predicted the presence of TMDp (OR = 5.783; *p* = 0.013) and suggested that the minor allele may have altered the binding affinity of the PAX6 transcription factor, potentially affecting the biological activity of opiorphin [14]. Therefore, the current findings contribute to further elucidating the role of this SNP genotype in differentiating TMDp subtypes. Another study that stratified participants based on TMDp subtypes investigated polymorphisms in genes encoding serotonin receptors (*HTR2A* and *HTR3A*) and found that carriers of the minor allele experienced higher pain intensity in patients with myalgia [27]. 

In addition to genetic influence, both investigated psychological characteristics (anxiety and depression) were significant risk factors for TMDp myalgia (OR = 3.510, *p* = 0.040; OR = 3.135, *p* = 0.036, respectively). These findings are consistent with those of Viñals Narváez et al., who also reported elevated anxiety levels and reduced coping ability in patients with the myalgia subtype of TMDp [28]. The interplay between pain processing and emotional distress is well documented in chronic pain research and may influence both the perception and chronification of pain symptoms [29,30]. However, the exact cause–consequence relationship has yet to be elucidated.

In addition to psychological influences, biological pathways involving inflammatory mediators may also play a role in chronic TMD pain. Interleukin-8, encoded by *CXCL8*, is a proinflammatory cytokine previously associated with chronic pain [31]. Elevated IL-8 levels have been reported in saliva, synovial fluid, and muscle tissue of TMD patients compared to healthy controls [32,33,34]. It is hypothesised that such cytokines may influence pain perception not through direct tissue damage, but by modulating neuroinflammatory pathways and central sensitisation [6]. This could explain why, despite a known role in pain biology, the SNPs within *CXCL8* did not emerge as significant predictors of TMDp subtypes in this study.

This study has certain limitations. Although we included 85 TMDp patients and 85 healthy controls, a larger sample size would provide stronger conclusions. Another limitation is the gender imbalance, with a predominance of females reflecting differences in TMDp prevalence and psychological characteristics. To address this, we assessed whether the odds ratios (ORs) remained significant after adjusting the analyses for other risk factors and covariates, such as sex. Another limitation is the use of questionnaires to assess oral behaviours and psychological characteristics. Although validated and part of the DC/TMD protocol, these are self-reported measures and may carry the risk of participants providing inaccurate responses.

Nevertheless, this study’s strengths include a case–control design and well-defined TMDp patients diagnosed according to the standardised and internationally accepted DC/TMD protocol by calibrated and experienced TMD experts. Furthermore, in collaboration with experts in the field of genetics, we established a protocol that could be utilised in future studies exploring the role of genetic background in orofacial pain disorders.

Despite growing evidence that genetic variation influences numerous biological pathways, identifying reliable biomarkers for chronic pain disorders remains a major challenge. Nonetheless, our findings support the relevance of incorporating genetic and psychological screening in the characterisation of chronic TMD pain, and highlight the need for continued research to validate SNP-based biomarkers as tools for clinical diagnosis and risk stratification.

## Figures and Tables

**Table 1:** Frequency of Oral Behaviours and Psychological Characteristics Among TMDp Subtypes and Healthy Controls.

Variables		Arthralgia		Myalgia	
		TMDp (*n* = 74)	Controls (*n* = 85)	TMDp (*n* = 55)	Controls (*n* = 85)
OBC-tot	Mean (SD)	29.73 (11.21)	25.92 (7.26)	28.91 (11.25)	25.92 (7.26)
	*p* ^a^	**0.011**		0.057	
OBC-daytime	Mean (SD)	24.88 (10.49)	21.64 (6.85)	23.73 (10.86)	21.64 (6.85)
	*p* ^a^	**0.021**		0.164	
OBC-nighttime	Mean (SD)	5.15 (2.27)	4.34 (1.76)	5.47 (2.14)	4.34 (1.76)
	*p* ^a^	**0.013**		**0.001**	
GAD-7	Mean (SD)	4.86 (4.12)	4.06 (3.48)	4.84 (4.03)	4.06 (3.48)
	*p* ^a^	0.184		0.228	
PHQ-9	Mean (SD)	5.70 (4.88)	4.60 (3.59)	6.04 (4.82)	4.60 (3.59)
	*p* ^a^	0.104		**0.046**	

Abbreviations: TMDp, pain-related temporomandibular disorders; OBC-tot, Oral Behavioural Checklist-total score; OBC-daytime, Oral Behavioural Checklist for daytime oral behaviours; OBC-nighttime, Oral Behavioural Checklist for nighttime oral behaviours; GAD-7, Generalised Anxiety Disorder-7; PHQ-9, Patient Health Questionnaire-9; *p*, *p*-value; SD, standard deviation; *n*, number of participants. Significant values are displayed in bold. ^a^ Independent samples *t*-test.

**Table 2:** Distribution of SNP Genotypes in the *OPRPN* (Recessive Model) and *CXCL8* (Dominant Model) Genes Among Pain-Related TMD Subtypes and Healthy Controls.

Variables	Arthralgia				Myalgia			
	TMDp (74)		CTR (85)		TMDp (55)		CTR (85)	
rs1387964 (*OPRPN*) Alleles: Wild-T Minor-C	CC	CT + TT	CC	CT + TT	CC	CT + TT	CC	CT + TT
*n* (%)	9 (12.2)	65 (87.8)	3 (3.5)	82 (96.5)	8 (14.5)	47 (85.5)	3 (3.5)	82 (96.5)
*p*-value ^a^	**0.040**				**0.018**			
rs2227306 (*CXCL8*) Alleles: Wild-C Minor-T	CC	CT + TT	CC	CT + TT	CC	CT + TT	CC	CT + TT
*n* (%)	24 (32.4)	50 (67.6)	39 (45.9)	46 (54.1)	18 (32.7)	37 (67.3)	39 (45.9)	46 (54.1)
*p*-value ^b^	0.084				0.122			
rs2227307 (*CXCL8*) Alleles: Wild-T Minor-G	TT	TG + GG	TT	TG + GG	TT	TG + GG	TT	TG + GG
*n* (%)	22 (29.7)	52 (70.3)	38 (44.7)	47 (55.3)	18 (32.7)	37 (67.3)	38 (44.7)	47 (55.3)
*p*-value ^b^	0.052				0.158			

Abbreviations: TMDp, pain-related temporomandibular disorders; CTR, pain-free control group; *OPRPN*, opiorphin gene; *CXCL8*, C-X-C motif chemokine ligand 8 gene; *n*, number of participants. Significant values are displayed in bold. ^a^ Fisher Exact Test; ^b^ Chi-Squared Test.

**Table 3:** Logistic Regression Models for Predictors of Pain-Related TMD Subtype: Arthralgia.

Model I	B	S.E.	*p*	OR	95%CI
rs2227307 (*CXCL8*) (homozygous TT_0; heterozygous TG + GG_1)	0.596	0.366	0.104	1.815	0.885–3.719
age	0.051	0.020	**0.009**	1.052	1.031–1.093
sex (male_0; female_1)	1.706	0.562	**0.002**	5.506	1.829–16.578
GAD-7 (low_0; high_1)	1.161	0.595	0.051	3.194	0.995–10.251
OBC (low_0; high_1)	−0.186	0.369	0.614	0.830	0.403–1.711
**Model II**					
rs2227307 (*CXCL8*) (homozygous TT_0; heterozygous TG + GG_1)	0.647	0.369	0.080	1.910	0.926–3.939
age	0.049	0.020	**0.014**	1.050	1.010–1.091
sex (male_0; female_1)	1.842	0.575	**0.001**	6.308	2.043–19.476
PHQ-9 (low_0; high_1)	1.004	0.551	0.069	2.728	0.926–8.040
OBC (low_0; high_1)	−0.223	0.375	0.551	0.800	0.384–1.667

Abbreviations: *CXCL8*, C-X-C motif chemokine ligand 8 gene; GAD-7, Generalised Anxiety Disorder-7; PHQ-9, Patient Health Questionnaire-9; OBC, Oral Behavioural Checklist-total score; B non-standardised regression coefficient; S.E., standard error; *p*, *p*-value; OR, odds ratio; CI, confidence interval. Significant values are displayed in bold.

**Table 4:** Logistic Regression Models for Predictors of Pain-Related TMD Subtype: Myalgia.

Model I	B	S.E.	p	OR	95%CI
rs1387964 (*OPRPN*) (homozygous CC_1; heterozygous CT + TT_0)	1.893	0.744	**0.011**	6.638	1.544–28.540
age	0.043	0.021	**0.038**	1.044	1.002–1.087
sex (male_0; female_1)	1.150	0.540	**0.033**	3.158	1.095–9.105
GAD-7 (low_0; high_1)	1.256	0.612	**0.040**	3.510	1.057–11.657
OBC (low_0; high_1)	−0.289	0.394	0.464	0.749	0.346–1.623
**Model II**					
rs1387964 (*OPRPN*) (homozygous CC_1; heterozygous CT + TT_0)	1.908	0.757	**0.012**	6.738	1.530–29.680
age	0.043	0.021	**0.040**	1.044	1.002–1.088
sex (male_0; female_1)	1.321	0.549	**0.016**	3.748	1.279–10.984
PHQ-9 (low_0; high_1)	1.143	0.545	**0.036**	3.135	1.077–9.125
OBC (low_0; high_1)	−0.311	0.396	0.433	0.733	0.337–1.593

Abbreviations: *OPRPN*, opiorphin gene; GAD-7, Generalised Anxiety Disorder-7; PHQ-9, Patient Health Questionnaire-9; OBC, Oral Behavioural Checklist-total score; B, non-standardised regression coefficient; S.E., standard error; p, *p*-value; OR, odds ratio; CI, confidence interval. Significant values are displayed in bold.

## Data Availability

Data are contained within the article. Any data that may be relevant to this research are available from the corresponding author upon reasonable request.
